# A Pediatric Patient with Refractory Seizures and a Mesial Temporal Lobe Lesion

**DOI:** 10.3389/fneur.2015.00129

**Published:** 2015-06-05

**Authors:** Marisa McGinley, Haiyan Chen, Douglas Anderson, Jorge Asconape, José Biller

**Affiliations:** ^1^Department of Neurology, Loyola University Medical Center, Maywood, IL, USA; ^2^Department of Pathology, Loyola University Medical Center, Maywood, IL, USA; ^3^Department of Neurosurgery, Loyola University Medical Center, Maywood, IL, USA

**Keywords:** arteriovenous malformation, seizures, mesial temporal lobe lesion, magnetic resonance imaging

## Abstract

A 12-year-old adolescent presented with refractory seizures and was found to have a mesial temporal lobe lesion. The patient underwent biopsy and was diagnosed with an arteriovenous malformation. Supratentorial lesions in the pediatric population can have a large variety of underlying etiologies, which can be challenging to differentiate on neuroimaging. In this report, we discuss the key features on MRI of several neoplastic, vascular, and infectious processes that can aide in the diagnosis.

## Clinical Case

A 12-year-old adolescent presented to his primary care physician with new onset staring episodes. His family stated the patient would be unresponsive during these episodes and would make a “wringing” motion with his hands. The episodes lasted about 1 min and were not preceded by an aura. After the episodes, the patient felt tired. At the time of presentation, the episodes were occurring daily. He was initially started on carbamazepine by his primary care physician who thought the episodes were consistent with seizures and he was referred to neurology. His past medical history was unremarkable. He was a full-term birth and had normal development. He was performing well in school and involved in several athletic activities.

**Figure 1:**
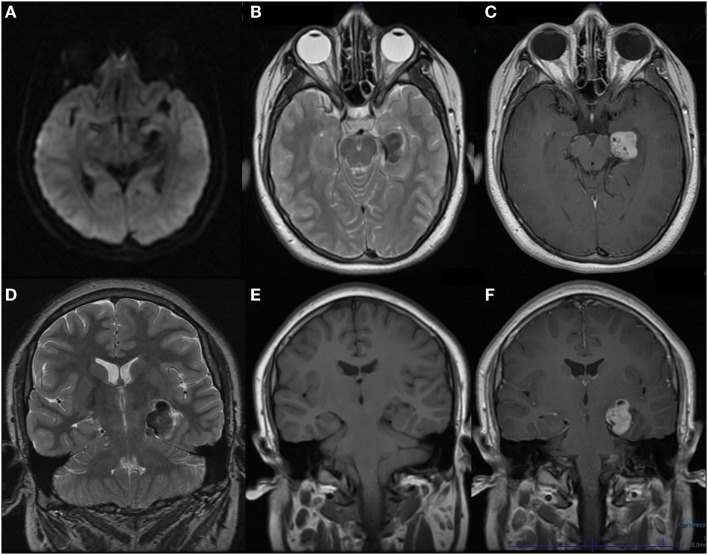
**MRI brain with and without contrast obtained at onset of seizures demonstrating left mesial temporal lobe lesion**. **(A)** Axial DWI **(B)** Axial T2 **(C)** Axial T1 post-contrast **(D)** Coronal T2 **(E)** Coronal T1 pre-contrast **(F)** Coronal T1 post-contrast.

On presentation to neurology, it was thought his episodes were consistent with complex partial seizures. His neurologic examination was unremarkable. His seizures were well controlled on the carbamazepine, which was continued and an EEG and MRI brain with and without contrast were ordered. The EEG was unremarkable, but the MRI brain showed a left mesial temporal lobe lesion (Figure 1). At this time, he was referred to neurosurgery for further evaluation and management. There was concern that this lesion was consistent with an underlying neoplasm, specifically a glial tumor, ependymoma, or dysembryoplastic neuroepithelial tumor (DNET). He underwent a left pterional craniotomy with partial resection of the left temporal lobe lesion. The pathology from this lesion was not diagnostic for any specific pathology. It demonstrated a firm collagenized reactive process that could represent an infectious, neoplastic, or reaction to a vascular malformation. He did well post-operative. He continued to have occasional seizures every couple of months and levetiracetam was added. After the addition of levetiracetam, he had better control, but over the next several years both his carbamezepine and levetiracetam were increased after an increase in seizure activity. The patient continued to have an annual MRI brain with and without contrast that demonstrated no change in the lesion. Five years after the initial surgery, the patient continued to have seizures (approximately 2–3/month) and repeat surgery was discussed for possible better seizure control. He underwent a second left temporal craniotomy for resection of the remaining mesial temporal lobe lesion. His immediate post-operative course was complicated by right-sided hemiplegia with left-sided intraventricular hemorrhage and an ischemic stroke affecting the left thalamus and left middle cerebral peduncle. Pathology from this biopsy was consistent with a congenital arteriovenous malformation (AVM) with secondary leptomeningeal fibrosis. He was discharged to an inpatient rehabilitation facility and he had partial recovery of strength on his right side to the point and he was able to ambulate with a cane. He was seen in follow-up and at 28 months post-surgery his levetiracetam was discontinued over 6 weeks and at 34 months post-surgery the carbamazepine was discontinued over 12 weeks. He has remained seizure free over the 3 years since surgery.

## Pathology

**Figure 2:**
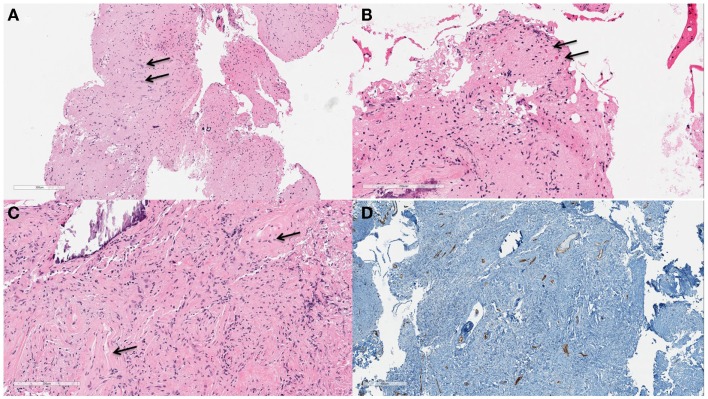
**Left temporal lobe lesion biopsy demonstrating hypercellular cortex (A)**. Pyramidal neurons are readily appreciated [**(B)**, arrows] on higher power (100×). At 200× magnification gliosis and focal brightly eosinophilic Rosenthal fiber formation [**(C)**, arrows] are appreciated. A CD34 immunostain **(D)** highlights the endothelial cells of the intraparenchymal vessels which are slightly increased in number.

A biopsy of the left mesial temporal lobe lesion was analyzed (Figure 2). On microscopic examination, the cortex was slightly hypercellular. There was evidence of gliosis with focal brightly eosinophilic Rosenthal fiber formation. Additionally, there was evidence of abnormal vessels with thickened walls within the brain parenchyma and one with extensive calcification. The GFAP and CD34 immunostain demonstrated cortex with reactive gliosis and intraparenchymal thin-walled as well as thickened blood vessels with intervening brain tissue. The leptomeninges showed extensive fibrosis and mineralization. A synaptophysin stain showed positive process in the neuropil, but is negative in the neuronal cell bodies. A Ki-67 proliferation index was <1%. These histological and immunohistochemical features were most consistent with an AVM with secondary leptomeningeal fibrosis.

## Discussion

This case demonstrates the difficulty in distinguishing neoplastic, vascular, and infectious lesions on imaging. Additionally the patient presented in this case had an initial biopsy that was inconclusive, which further supports the importance of gleaning as much information as possible from imaging studies. In this report, we provide a brief summary of the classic imaging characteristics of a variety of neoplastic, vascular, and infectious etiologies and discuss how this may have helped with the initial work-up and diagnosis of this patient.

In the pediatric population, supratentorial cortical based tumors represent 25–40% of all brain tumors (1). Most of these tumors are low grade resulting in a slow progression with a mean latency to diagnosis of 8 months (1). Table 1 provides a summary of the typical imaging characteristics of the low- and high-grade tumors often found in pediatric patients. In general, low-grade tumors appear more homogeneous, whereas high-grade tumors often have a more heterogeneous appearance because of cystic, hemorrhagic, and necrotic portions. Additionally, most tumors are hyperintense on T2/FLAIR. The patient presented in this case had a lesion that was primarily hypointense on T2, which is in contrast to most neoplastic lesions, but could be consistent with a low-grade pilocytic astrocytoma, pleomorphic xanthoastrocytoma, or meningioma. Of these three tumor types, the only one that demonstrates strong contrast enhancement, as in the patient reported, is a meningioma. The patient’s lesion did not appear dural based, but this is not always readily apparent on imaging.

**Table 1:** **Summary of characteristic imaging features of supratentorial tumors seen in the pediatric population (1–3)**.

Tumor type	Common location	T2/FLAIR	T1	T1 post-contrast	Mass effect
Pilocytic astrocytoma	Cerebellum, brainstem, optic nerve, thalamus	Hyperintense (cystic portion), Iso/hypointense (solid)	Iso/hypointense	Cyst wall may have ring like enhancement	None
Fibrillary astrocytoma	Cerebellum, brainstem, optic nerve, thalamus	Hyperintense	Hypointense	No enhancement	Minimal
Pleomorphic xanthoastrocytoma	Parietal/temporal	Isointense	Isointense	Positive enhancement	Extensive
Gangliogliomas	Frontal and temporal	Hyperintense	Iso/hypointense	20% Enhance	Minimal
Dysembryoplastic neuroepithelial	Frontal/temporal	Well defined hyperintense rim	Iso/hypointense	Rarely enhances	Minimal
Oligodendrogliomas	Cerebral hemispheres	Hyperintense	Iso/hypointense	Occasional enhancement in higher grades	Minimal
Ependymomas	Fourth Ventricle, less likely third or lateral ventricle	Hyperintense or heterogeneous	Hypointense	Mixed, solid portions will enhance	Yes
Meningioma	Frontoparietal parasagittal convexities, falx, tentorium cerebelli, sphenoid wings, olfactory groove, and tuberculum sella	Isointense or heterogeneous	Iso/hypointense	Positive enhancement	Variable
Glioblastoma	Cerebral	Hyperintense	Hypointense	Ring-like	Significant
Gliomatosis cerebri	Diencephalon/basal ganglia			50% Incomplete enhancement	Minimal
Primitive neuroectodermal	Fronto/parietal	Hyperintense (cystic/necrotic)	Hyperintense (hemorrhage)	Positive enhancement	Minimal
Atypical teratoid/rhabdoid	Vermis/cerebellopontine angle, occasional cortical	Isointense or slightly hyperintense	Heterogeneous	Positive enhancement	
Lymphoma	Anywhere, classically periventricular	Heterogeneous	Hypo/isointense	Positive enhancement	Minimal

Infectious and vascular etiologies would also be on the differential for this patient. Infectious etiologies to consider are an abscess and limbic encephalitis. Abscesses can have a range of characteristics on MRI depending on the age of the lesion. Early in the course it will have an ill-defined shape with patchy enhancement (4). As the lesion matures, it will become well defined with a thin rim like enhancement (4). On T1 the lesion will initially be hypointense or isointense and with time will develop a hyperintense rim. It will always appear hyperintense on T2, but with progression will develop a hypointense rim (4). Initially, the lesion will have some mild restricted diffusion and with time will become very bright centrally on diffusion imaging. Limbic encephalitis may have been considered given the mesial temporal lobe location with a clinical history of seizure, but this process is ill-defined and hyperintense on T2 imaging.

The primary vascular lesions that would be considered are cavernous malformations and AVM. The majority of cavernous malformations occur supratentorial and typically present in the third through fifth decades of life, rarely in childhood. Cavernous malformations can have a wide range of radiological appearances on imaging depending on the lesion type (I–IV) (5). A high grade lesion will be hyperintense on T1 with a mixed signal on T2, whereas a low grade lesion will be iso/hypointense on T1 and T2 or poorly visualized (5). These lesions typically have a “popcorn” appearance and do not enhance with contrast. Arteriovenous malformations are an abnormal connection between an artery and vein with an intervening capillary bed. The classical type is a pial AVM with a glomerular or compact type nidus (6). On MRI, they have a classic “bag of worms” appearance secondary to flow voids. They are typically hypointense with a surrounding hyperintensity on T2/FLAIR and will enhance with contrast (7). Given these imaging characteristics the patient presented in this case did have imaging findings that were consistent with an AVM.

After reviewing this case, there are a few other ancillary tests that may have contributed to the diagnosis of this patient. When confronted with a lesion that has characteristics of multiple different underlying pathologies further studies that can help are vascular imaging (e.g., MRA, CTA, conventional angiogram) and functional/metabolic studies (e.g., MR spectroscopy and PET scans). MR spectroscopy identifies markers of cellular proliferation and certain profiles are suggestive of neoplastic lesions versus infection or ischemia (1, 2). Similarly 2-deoxy-2[^18^F]fluoro-d-glucose (FDG)–PET scans analyze the uptake of glucose, which is increased in malignant tumors. PET scans are especially helpful for distinguishing recurrent tumor from radiation necrosis (1). In the patient presented in this case these functional studies could have helped support a non-neoplastic underlying lesion. The limitation of these functional studies, especially FDG–PET, is that low grade neoplasms often have little activity, which can be misleading. There are several aminotracers available that may have some benefits over FDG when there is a concern for a low-grade tumor or non-neoplastic lesion. The primary benefit is that the uptake of amino acids in normal brain tissues is low, as opposed to the high uptake of glucose in normal brain tissue, which allows for the better identification of low grade tumors. L-[methyl-^11^C] methionine (MET) and ^18^F-fluroethyl-tyrosine (FET) are currently available options, but one limitation is that they are not commercially available at all institutions (8, 9). Additionally, MET has a very short half-life (20 min) requiring an in-house cyclotron facility (8, 9). Although these tracers are reported to be more sensitive and specific than FDG–PET, there are still reports of increased uptake in the settings of inflammatory cells, reactive glial tissue, hematomas, ischemia, abscesses, and acute demyelination, which must be kept in mind. Although these ancillary tests may not give a definitive diagnosis, they will contribute to the larger clinical picture. In the case presented, these tests may have directed the treatment provided more toward a non-neoplastic lesions, specifically a vascular lesion, which may have changed the surgical approach.

## Conclusion

This case of an underlying AVM resulting in a refractory seizure disorder highlights the challenge in distinguishing neoplastic, infectious, and vascular lesions based on imaging. MRI can provide a great deal of information about a lesion and it is important to carefully analyze available imaging to obtain as much information as possible. Additionally, when uncertainty remains about diagnosis, it may be helpful to utilize other imaging modalities including vascular imaging, MR spectroscopy, and PET scans. Finally, when a diagnosis remains unclear, biopsy needs to be considered, but ultimately it is the entire work-up and clinical picture that will lead to a correct diagnosis.

## Conflict of Interest Statement

The authors declare that the research was conducted in the absence of any commercial or financial relationships that could be construed as a potential conflict of interest.
